# Influence of internal variability on future changes in surface wind speed in China with two large ensemble simulations

**DOI:** 10.1371/journal.pone.0319210

**Published:** 2025-03-21

**Authors:** Ling Yu, Hong Cao, Liang Yue

**Affiliations:** State Grid Songjiang Power Supply Company, Shanghai, China; Texas A&M University, UNITED STATES OF AMERICA

## Abstract

Wind energy, as one of the renewable energy sources, plays a crucial role in the global energy system’s transition to clean energy. China possesses vast and widely distributed wind energy resources, and in recent years, it has rapidly developed and begun large-scale commercial utilization. Therefore, studying changes in surface wind speeds (SWSs) is highly important for wind energy development in China. This study utilizes two initial condition large ensemble simulations to project future changes in SWSs over China. The two sets of initial large ensemble models used are CanESM2-LE and CESM1-LE. By comparing the results from these two large ensemble models, the influence of internal variability of the climate system on SWSs in China are studied. Both models can effectively reproduce the climatological spatial distribution of SWSs in reanalysis. Results from both models indicate that external forcing leads to an increase in winter SWSs in eastern China, while SWSs decreases in the southeastern coastal areas and southwestern Tibet. In summer, SWSs exhibits a pattern of decrease in the north and increase in the south. The magnitude of wind speed changes is greater in winter than in summer. Additionally, as the projected period extends, the magnitude of these changes intensifies. The research results can provide a scientific basis for the future planning of wind power deployment.

## 1. Introduction

Wind is an important meteorological element closely related to phenomena such as air pollution and dust storms, exerting a widespread and significant influence[1,2]. Wind energy resources, a clean renewable energy source, result from uneven distribution of solar radiation received at the Earth’s surface, causing uneven heating of the atmosphere and horizontal air movement, thereby generating kinetic energy from air flow. Wind energy represents a form of solar energy conversion with low development and utilization costs, making it one of the most promising renewable energy sources. China boasts a wide distribution and abundant development potential of wind energy resources[3], playing a pivotal role in the country’s electricity supply. With the development of China’s wind power industry, the sustainable utilization of wind energy resources under a warming climate has garnered considerable attention, leading to advancements in wind resource assessment[4].

However, recent studies indicate a general weakening trend in surface wind speeds (SWSs) across most regions of China, with the highest rates of decline occurring in the northwest, north, and northeast—areas rich in wind energy resources[5,6]. In fact, the decrease in SWSs is a global phenomenon [7]. Global surface SWSs have shown a significant weakening trend over the past four decades, this decline has not been uniform but rather exhibits notable decadal variability. The decline in SWSs hampers the dispersion of atmospheric pollutants, increases the frequency of haze days, reduces occurrences of dust storms, and diminishes the amount of radiation reaching the Earth’s surface, directly or indirectly affecting regional water cycle processes[8]. A turning point around 2010 where the long-term trend in global average SWSs shifted from significant weakening to significant strengthening [7]. The earliest appearance of this turnaround in SWSs was observed in Asia, with China’s SWSs serving as the primary data source for the Asia [9]. Compared to other global regions, the changes in SWSs across China exhibit a more complex pattern, characterized by distinct regional differences [6,10,11]. There are many factors that can explain the changes in global SWSs [12], including increased vegetation and development of urbanization, which enhances surface friction on winds [13]; the Arctic amplification effect, which weakens the westerly winds [14]; and the internal variability of climate system [15], and changes in the observation instrument [16,17].

Climate models are useful tools for studying the climate change, with their simulation results serving as crucial data foundations for climate future projections and climate change risk assessments [18,19]. Many studies have utilized the CMIP5 (Coupled Model Intercomparison Project Phase 5) models to investigate the changes in SWSs. The uncertainties in future projections from CMIP5 multi-model ensembles primarily stem from three sources: 1) uncertainty in future emission scenarios, 2) model uncertainty, and 3) internal variability of the climate system. The internal climate variability often contributes more to uncertainty than other factors [20]. However, using the CMIP5 model framework to project the changes in SWSs may provide misleading information for regional-scale wind energy resource assessments. To improve the credibility of future changes in SWSs at regional scale, initial-condition large ensemble simulations have been developed in recent years. Each ensemble member experiences the same external forces but different initial conditions, reflecting the influence of internal variability among different members, while the ensemble mean reflects the response to external forcing [21]. Many studies have used large ensemble simulations to explore the contribution of internal variability and external forcing to the future climate change [22–24], However, understanding of SWSs changes over the China is relatively limited.

Therefore, this study uses large ensemble simulations to quantify the contributions of internal variability and external forcing to future changes in SWSs over China. The aim is to provide reliable projections of future SWSs over the region, offering a scientific basis for policy-making and risk assessment.

## 2. Data and methods

### 2.1. Model simulation datasets

The first large ensemble simulation is the second-generation Canadian Earth System Model Large Ensemble (CanESM2-LE), developed by the Canadian Centre for Climate Modelling and Analysis (CCCma), with a spatial resolution of 2.8° ×  2.8° [25]. It is a fourth-generation coupled global climate model combining the atmosphere-ocean physics model CanCM4 with land carbon model (CTEM) and ocean carbon model (CMOC). The CanESM2-LE was created by applying new random atmospheric perturbations in 1950 to the five members of the existing Coupled Model Intercomparison Project Phase 5 historical ensemble. Each historical run was then used to generate 10 ensemble members, resulting in a total of 50 members starting in 1950. All ensemble members were forced with the same historical factors, including observed explosive volcanoes, the solar cycle, greenhouse gases, aerosols, ozone, and land use during the historical period up to 2005. From 2006 to 2099, the simulations followed the Representative Concentration Pathway 8.5 (RCP8.5) scenario. We extended the historical simulation to 2017 using simulations under RCP8.5 scenarios, aligning it with the observed record.

The second ensemble with different initial conditions is the Earth System Model Large Ensemble (CESM-LE), composed of four independent modules simulating Earth’s atmosphere, ocean, land surface, sea ice, and a central coupler component with a spatial resolution of nearly 2.8° ×  2.8° [26]. Each member was subject to the same radiative forcing as used for the CanESM2-LE (i.e., historical forcing up to 2005, followed by RCP8.5 forcing through 2100). CESM generates large ensemble experiments at approximately 1° latitude-longitude horizontal resolution, and ensemble members are generated by initiating from a slightly perturbed initial temperature field within the rounding error range of each realization. The historical simulation for the period after 2005 (2006–2017] was extended using simulations under the RCP8.5 scenario. Employing two models allowed us to validate the robustness of the results. Model outputs were extracted exclusively for the China region.

The contribution of internal variability on the future projection of SWSs is explored by analyzing differences across ensemble members. For the future periods, we selected the mid-21st century (20546-2065) and the end of 21st century (2081–2100), each spanning 20 years. To ensure consistency with the future periods, the historical period of 1986–2005 was chosen. Finally, model uncertainty in the results is investigated by comparing the outcomes of two models.

### 2.2. Reanalysis dataset

We use SWSs from the European Centre for Medium-Range Weather Forecasts (ECMWF), the ERA5 reanalysis (ERA5) to evaluate the performance of the two large ensemble simulations for historical SWSs during winter and summer. The ERA5 is the latest reanalysis dataset produced by the ECMWF, providing a wide range of atmospheric, land-surface, and sea-state variables globally [27]. It is based on the Integrated Forecasting System (IFS) Cycle 41r2 model, which has a horizontal resolution of 31 km and a total of 137 vertical levels (with the model’s top at 0.01 hPa). The model also incorporates advanced representations of sub-grid scale processes, including a scheme for large-scale cloud and precipitation, prognostic variables for precipitating rain and snow, and an updated deep-convection scheme (see Hersbach et al., 2020 and references therein). In the surface layer (up to the lowest model level, approximately 10 m), the model applies Monin–Obukhov similarity theory to represent turbulent fluxes between the surface and the atmosphere. This study uses monthly surface wind speed data from ERA5.

### 2.3. Methods

#### 2.3.1. Mann-Kendall trend analysis.

We used the Sens’s slope to estimate the trend, which is a robust non-parametric statistical approach for trend estimation [28]. This method is known for its computational efficiency and insensitivity to measurement errors and outliers, making it particularly useful for trend analysis in long time series data.


$$\beta =\text{mean}\left(\frac{x_{j}-x_{i}}{j-i}\right),\forall j>i$$ (1)

In the formula, $x_{j}$ and $x_{i}$ represent the time series. A positive value of β indicates that the time series exhibits an increasing trend, while a negative value of β indicates a decreasing trend.

We used the Mann-Kendall Test to conduct the statistical significance, which is a non-parametric test. Its advantage lies in not requiring the data to follow a specific distribution and being robust against outliers. It is commonly used to analyze trends such as precipitation, runoff, temperature, and water quality.

For time series X, the statistic S for the Mann-Kendall trend test is computed as follows:


$$S=\overset{n-1}{\underset{i=1}{\sum }}\overset{n}{\underset{j=i+1}{\sum }}\mathrm{sgn}\left(X_{j}-X_{i}\right)$$ (2)

Where $X_{i}$ and $X_{j}$ are the i-th and j-th data values of the time series respectively, with i<j; n represents the length of the data sample; sgn is the sign function, defined as follows:


$$\text{sgn}\left(X_{j}-X_{i}\right)=\left\{\begin{array}{cc} 1 & \left(X_{j}-X_{i}\right)>0\\ 0 & \left(X_{j}-X_{i}\right)=0\\ -1 & \left(X_{j}-X_{i}\right)<0 \end{array}\right\}$$ (3)

When n ≥ 8, the statistic S approximately follows a normal distribution. Assuming no ties in the sequence, the mean E(S) is 0 and the variance $Var\left(S\right)=n\left(n-1\right)\left(2n+5\right)/18$. The standardized test statistic Z is calculated as follows:


$$Z=\left\{\begin{array}{cc} \frac{S-1}{\sqrt{V_{ar}\left(S\right)}} & S>0\\ 0 & S=0\\ \frac{S+1}{\sqrt{V_{ar}\left(S\right)}} & S<0 \end{array}\right\}$$ (4)

In a two-sided trend test, for a given confidence level (significance level) α, if $\left|Z\right|\geq Z1-\alpha /2$, the null hypothesis H0 (H0: no monotonic trend) is rejected. Thus, at the significance level α, the time series data shows a significant upward or downward trend. The absolute value of Z being greater than or equal to 1.96 corresponds to passing significance tests at the 95% confidence levels. We calculate the trend during the historical period (1961-2005), due to sufficient number of samples of SWSs.

## 3. Results

### 3.1. Characteristics of SWSs in Reanalysis and Simulations

**Fig 1:**
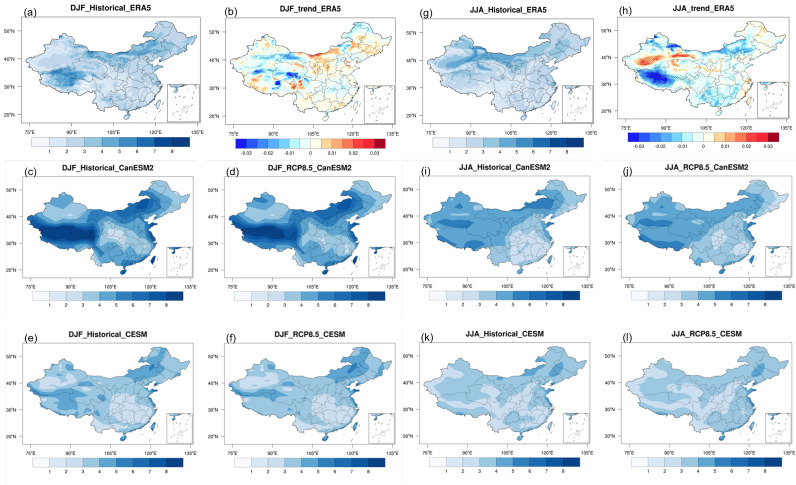
Spatial distributions of SWSs based on ERA5 reanalysis data for winter ( a, b**) and summer (**g, h**), including the climatology for 1986–2005 (**a, g**) and long-term trends for 1961–2005 (**b, h**).** **Additionally, spatial distributions of climatology for the historical period (1986–2005) and the late 21st century under the RCP8.5 scenario (2081–2100) are presented for two models, CanESM5 (**c, d, i, j**) and CESM1 (**e, f, k, l**), for both winter and summer. (Made with Natural Earth. Free vector and raster map data @ naturalearthdata.com.).**

Fig 1 shows the spatial distribution of historical climatology and trends in SWSs for DJF and JJA across China from historical period (1986-2005) from reanalysis (Fig 1a, b, g, h), as well as the historical period and future period (2081-2100) under RCP8.5 scenario from CanESM2 and CESM. The spatial distribution of climatology from reanalysis indicates that during the historical period, due to the larger temperature differences between low and high latitudes, as well as the difference between land and sea in winter compared to summer, SWS over the China region are stronger in winter than in summer [29]. The maximum SWSs were concentrated in three main regions in both DJF and JJA: the Sichuan-Tibetan region (including Tibet Autonomous Region, Qinghai Province, and western Sichuan), Inner Mongolia and eastern China, and the coastal areas of southern China. The distribution of SWSs is closely related to variations in terrain. The Qinghai-Tibet region has high elevation and open terrain, making it susceptible to influences from the westerlies and northwest air currents, resulting in higher SWSs. The border area between Inner Mongolia and China’s central region has relatively flat terrain, making it prone to influences from cold surge and cyclonic winds, also leading to higher SWSs. Eastern China, being a coastal region, is influenced by both maritime and terrestrial factors, including the winter monsoon, contributing to higher SWSs in this area.

The two climate models can generally capture the spatial structure of the climatology of SWSs in DJF (Fig 1 c, e) and JJA (Fig 1 i k) in reanalysis. However, there are significant differences between the simulation of the two models. For instance, CanESM2 (Fig 1c) shows a localized maximum DJF SWSs (>8m/s) near the border of Tibet Region and Qinghai Province, which is less pronounced in CESM (Fig 1e). Additionally, CanESM2 exhibits greater spatial variability in SWSs across China, ranging from 1 to 8 m/s, whereas CESM demonstrates spatial uniformity with speeds ranging from 2 to 4 m/s. The magnitude of simulated SWSs in CESM is smaller compared to CanESM2. Regarding to the climatology of SWSs in future simulations in DJF (Fig 1d, f) and JJA (Fig 1 j, l), the spatial pattern is very similar to that in historical period, with the only difference being in the magnitude.

The regions with an increase in SWSs during the winter are primarily located in Northeast China and North China, with the most pronounced increasing trend observed in Inner Mongolia. In contrast, a decreasing trend is seen in the western regions, with the largest decrease occurring in the Tibetan Plateau. The trend changes in summer are more pronounced, especially in the western regions. Notably, the southwestern and northwestern parts of Tibet show a significant decrease in SWSs, while a marked increase is observed in the areas between these two regions. In the eastern part of China, a strong decreasing trend is evident in the northern areas, while the trend changes weaken in other regions. Our results are consistent with previous studies [12,30].

**Fig 2:**
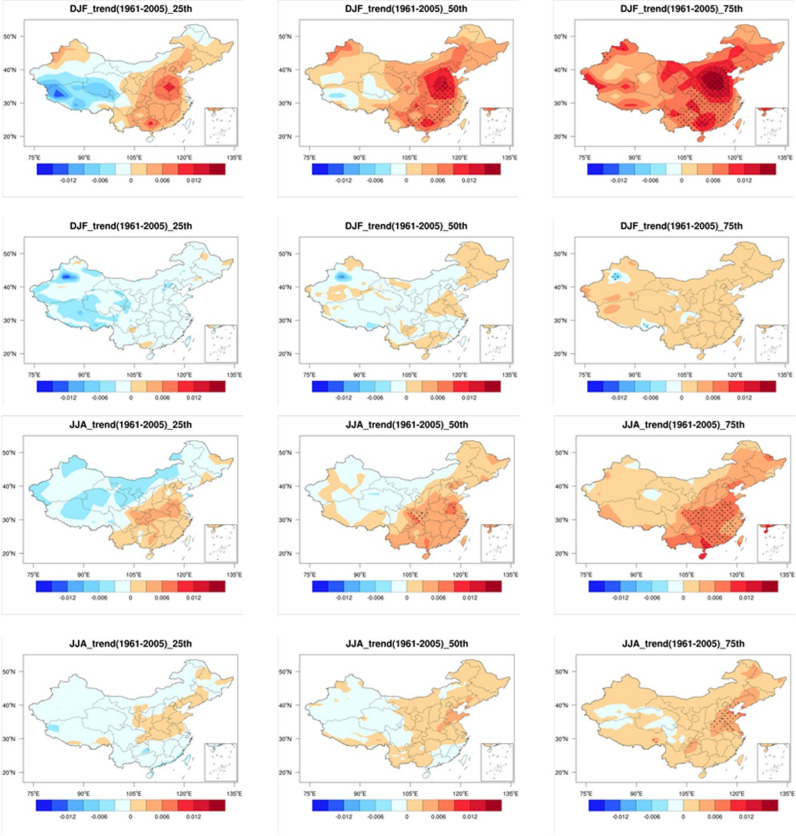
Spatial distributions of the trend changes in SWSs in winter and summer based on two large ensemble simulations, CanESM5 and CESM1, for the period 1961–2005. The 25th, 50th, and 75th percentiles are shown. Units: m/s/year. (Made with Natural Earth. Free vector and raster map data @ naturalearthdata.com.).

Since differences among members of the large ensemble simulation represent the contribution of internal variability, Fig 2 illustrates the influence of internal variability on the SWSs trend in DJF and JJA. It shows the spatial pattern of the 25th, 50th, and 75th percentiles of SWS trend from 50 members of CanESM2 and the 40 members of CESM. It is noteworthy that CanESM2 and CESM exhibit considerable differences in both spatial patterns and magnitudes of DJF and JJA SWSs trends, indicating the important influence of internal variability on SWSs over China.

The DJF SWS trend in eastern China show an increasing trend across different members. This indicates a strong influence of external forcing on increasing SWSs in eastern regions. However, in western China, the 25th, 50th, and 75th percentiles of SWSs gradually shift from a weak decreasing trend to an increasing trend, suggesting that internal variability played an dominate role on SWSs changes in these regions. In contrast, there are substantial differences in SWSs trend among different percentiles in CESM. The SWSs shift from a decrease in the 25th percentile to an increase in the 75th percentile. This indicates that, relative to CanESM2, the influence of internal variability on SWSs trends in CESM is more pronounced.

For SWSs in JJA, trends across different members are relatively consistent in CanESM2, showing an increase. However, in western China, there is a quite large difference among members, indicating that internal variability has a greater influence in this region than in the eastern China. For CESM, only part areas in eastern China exhibit changes similar to those of CanESM2, while the 25th and 75th percentile trends in other regions are completely opposite.

In summary, DJF and JJA SWSs in eastern China are increasing, likely due to the influence of external forcing. In contrast, SWSs changes in western China are more strongly affected by internal variability. A comparison with the observed trend distribution reveals notable differences between the model and reanalysis. The observed trends are primarily influenced by the combined effects of external forcing and internal variability, resulting in pronounced spatial heterogeneity. In contrast, the model-simulated trends exhibit lower spatial variability. For instance, the observed trends show greater magnitudes in the western China and smaller ones in the eastern China, whereas the model produces the opposite pattern. Discrepancies are even apparent in the direction of the trends.

These differences between the model and reanalysis arise partly because the model cannot fully reproduce the contributions of internal variability and external forcing to the trends, and partly due to insufficient model resolution to capture the complex terrain in western regions [31,32]. However, this study focuses primarily on the contributions of external forcing and internal variability. To this end, simulations from two large ensemble simulations are used to investigate the impact of internal variability on the projections of SWSs over China.

### 3.2. Influence of Internal Variability on Future SWSs Changes

#### 3.2.1. SWSs changes in the middle of 21 century (2046-2065).

**Fig 3:**
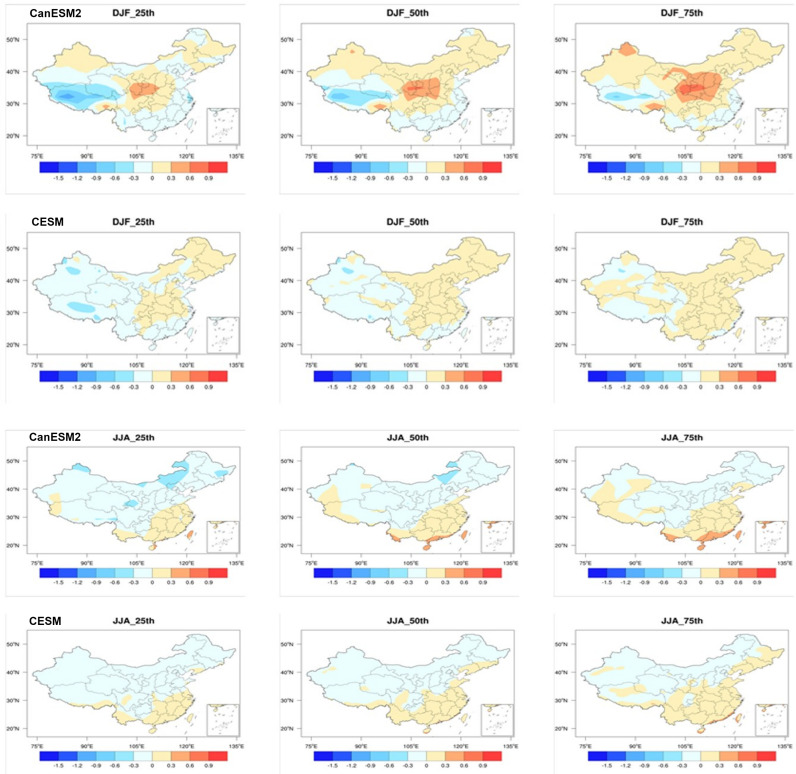
Spatial distributions of SWSs changes in winter and summer for the mid-21st century (2046–2065) relative to the historical period (1986–2005) based on CanESM5 and CESM1. The 25th, 50th, and 75th percentiles are shown. Units: m/s. (Made with Natural Earth. Free vector and raster map data @ naturalearthdata.com.).

Next, we studied the future changes in SWSs over China during the mid-century (2046-2065) and end of century (2081-2100) under RCP8.5 scenario. Fig 3 shows the spatial pattern of changes in DJF and JJA SWSs during the mid-century relative to historical periods (1986-2005) across China. We also provide the 25th, 50th, and 75th percentiles from the members to reflect the influence of internal variability on future changes in SWSs.

For DJF, it can be observed that the results from both models consistently show that SWSs decreases in the southwestern Tibetan Plateau and the eastern coastal regions, while it increases in the central region. This spatial pattern of changes is likely driven by external forcing rather than the internal variability, due to that that the different members exhibit similar changes.

The 50th percentile reflects changes driven by external forcing. In the central region, the increase in SWSs can exceed 0.3 m/s in CanESM2, whereas in other regions with increased SWSs, the changes remain below 0.3 m/s. In the southwestern Tibetan Plateau, the decrease in SWSs can also exceed 0.3 m/s, while in the coastal areas of southern China, the reduction in SWSs does not exceed 0.3 m/s. However, the magnitude of changes in CESM is smaller compared to CanESM2. For instance, the changes across the entire China region do not exceed 0.3 m/s. The differences between the two models stem from variations in resolution, disparities in the parameterization processes, and differences in the models’ responses to external forcing in simulating regional SWSs changes.

For JJA, both models show an increase in SWSs in southeastern China, while a decrease is observed in other regions. The magnitude of summer changes is weaker compared to winter, primarily because the latitudinal temperature gradient is greater in winter than in summer, resulting in generally stronger SWSs during winter. Moreover, the variation in magnitude among different members is minimal, indicating that these changes are primarily driven by external forcing, with a relatively small contribution from internal variability.

As for the 50^th^ percentile of SWSs changes, in the southeastern region, the increase in SWSs does not exceed 0.3 m/s. However, in the southern coastal areas, the increase surpasses 0.3 m/s, and in parts of northern northeastern China, the decrease also exceeds 0.3 m/s. Compared to CanESM2, the magnitude of changes in CESM is smaller.

#### 3.2.2. SWSs changes in the end of 21 century (2081-2100).

**Fig 4:**
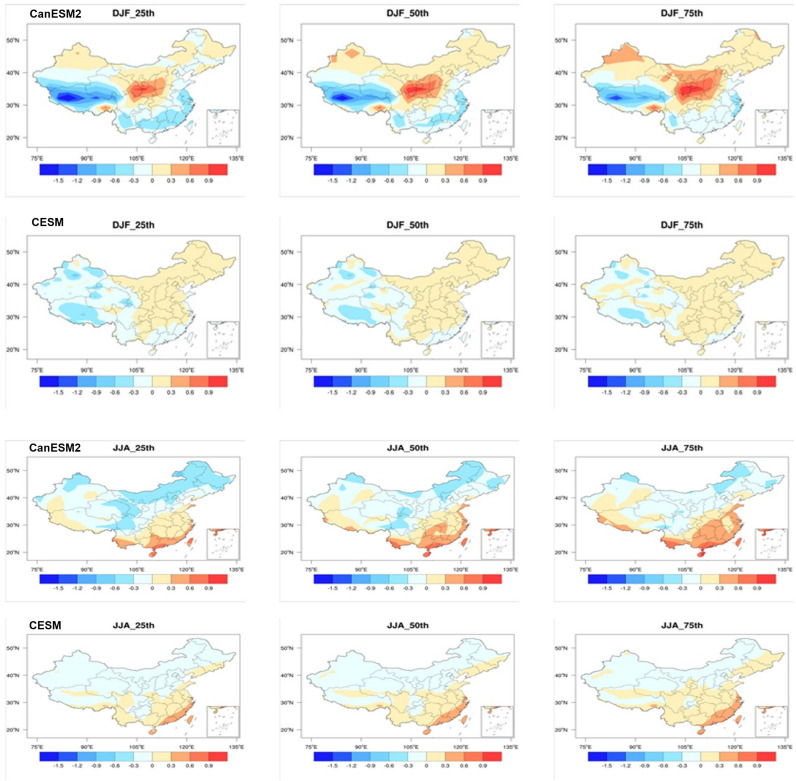
Spatial distributions of surface wind speed changes in winter and summer for the late 21st century (2081–2100) relative to the historical period (1986–2005) based on CanESM5 and CESM1. The 25th, 50th, and 75th percentiles are shown. Units: m/s. (Made with Natural Earth. Free vector and raster map data @ naturalearthdata.com.).

We provide an analysis of the changes in SWSs across China in DJF and JJA by the end of the 21st century (2081-2100) (Fig 4). While the overall spatial pattern remains relatively similar to the mid-term, the magnitude of these changes has become much more pronounced.

For DJF, in the CanESM2 model, external forcing leads to a decrease in SWSs in southwestern Tibet and the southeastern coastal regions of China, while increasing SWSs in central China. In the CESM model, external forcing results in an increase in SWSs in eastern China but a decrease in western China. This consistent pattern is observed across different ensemble members. Compared to the mid-term, the areas influenced by external forcing have expanded notably. For instance, in the mid-term, only parts of southwestern Tibet show consistent changes between the 25th and 75th percentiles of members, while in the end of century, the entire southwestern Tibet exhibits consistent changes between the 25th and 75th percentiles of members. Moreover, the increased SWSs amplitude is primarily attributed to external forcing. Therefore, as the projection period lengthens, the influence of internal variability decreases, while the impact of external forcing increases.

The 50th percentile results from CanESM2 indicate that external forcing leads to a decrease in SWSs exceeding 1.2 m/s in southwestern Tibet. However, the decrease in southeastern coastal regions is less than 0.6 m/s. In central China, the maximum increase in SWSs can exceed 0.9 m/s. The changes in CESM are relatively smaller. Except for a decrease exceeding 0.3 m/s in some western regions, the SWSs changes in other regions are all less than 0.3 m/s.

For JJA, the changes induced by external forcing result in increased SWSs in southern China and decreased SWSs in northern China. Both models exhibit a rather consistent pattern of change, with the only difference being that the magnitude of SWSs changes is weaker in CESM compared to CanESM2. Based on the 50th percentile results from CanESM2, the maximum increase in SWSs in southern China can exceed 0.9 m/s, with half of the region experiencing an increase of more than 0.6 m/s. In northern China, the largest decrease is primarily located in the northeast region, with a decrease exceeding 0.3 m/s. For CESM, except for the coastal areas in the south where the increase exceeds 0.3 m/s, the changes in SWSs in other regions are all less than 0.3 m/s.

### 3.3. Robust changes of SWSs in the future

**Fig 5:**
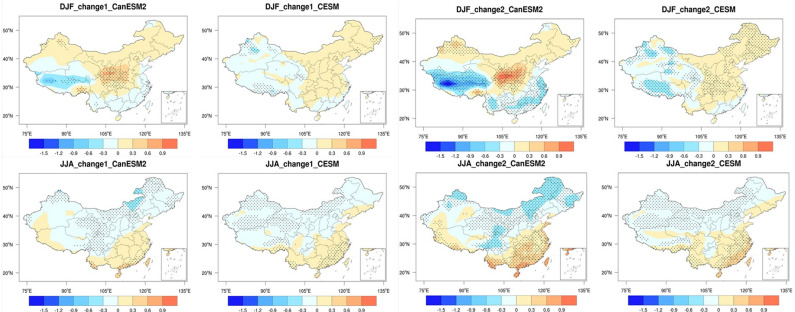
Spatial distributions of SWSs changes for the mid-21st century (2046–2065) and late 21st century (2081–2100) relative to the historical period (1986–2005) based on CanESM5 and CESM1. Dots indicate regions where the changes are statistically significant at the 5% level based on the t-test. (Made with Natural Earth. Free vector and raster map data @ naturalearthdata.com.).

Due to differences in physical parameters between the CanESM2 and CESM climate models, their projection in SWSs over China are not entirely consistent. To clearly illustrate and compare the differences between the two models, the future change signals defined by multi-member averages are shown in Fig 5. Significant tests are conducted to compare the consistency between the models, revealing consistent characteristics of future SWSs changes under external forcing.

The changes produced by the two models are markedly different. For instance, during the middle of 21 century period, CanESM2 shows a significant increase of SWSs in southeastern Tibet Autonomous Region for DJF, while CESM depicts a significant decrease in the same area. Similarly, in the northwestern region of China, CanESM2 shows an increase, while CESM exhibits a significant decrease. Additionally, in southwestern Tibet, although both models show a significant decrease, the magnitude of the decrease is larger in CanESM2 compared to CESM. For the JJA, both models consistently show a significant decrease in SWSs over northwestern China. However, due to factors such as model resolution and parameterization of physical processes, there is uncertainty regarding the exact regions where this decrease is most pronounced. CanESM2 simulations indicate a more significant decrease in SWSs in the eastern part of northwestern China, while CESM tends to simulate a more pronounced decrease in the western part.

By the end of 21st century, changes in SWSs across the country become more pronounced. For DJF, in CanESM2, there is a significant increase in SWSs over central China, while there is a significant decrease in SWSs over southwestern Tibet and Southern China. However, the results from CESM show considerable differences compared to CanESM2. Firstly, although there is also a significant decrease in central China in CESM, the magnitude of the decrease is much weaker. The decrease in CanESM2 can exceed 0.6 m/s, while the decrease in CESM does not exceed 0.3 m/s. Secondly, there is a significant increase in SWSs over northwestern China in CanESM2, but CESM shows a significant decrease. Finally, for southeastern China, CanESM2 shows a significant decrease, while CESM shows no significant change or even a slight increase.

For JJA, the results from CanESM2 indicate a significant increase in SWSs over southern China, with local increases exceeding 0.6 m/s in some areas. In contrast, there is a significant decrease in SWSs over northern China, with the largest decreases occurring in the northeast and parts of the central-western regions, exceeding 0.3 m/s. Compared to CanESM2, the changes in SWSs simulated by CESM are relatively smaller. The largest increases are primarily located in coastal areas of southern China, exceeding 0.3 m/s, while changes in other regions do not exceed 0.3 m/s. The two models show greater agreement in JJA than in DJF, both in the middle and end of 21^st^ century.

## 4. Conclusions and discussion

### 4.1. Conclusions

China’s energy industry has achieved significant progress in the process of energy transformation towards building a “clean, low-carbon, safe, and efficient” energy system. In facilitating the realization of carbon peaking and carbon neutrality goals, new energy, especially renewable energy, has considerable potential to tap into. The future changes in SWSs in China are crucial for evaluating wind energy resources. Due to considerable uncertainties in previous CMIP5 multi-model future projections, here we used two initial condition large ensemble simulation (CanESM2 and CESM) to assess the impacts of internal variability and external forcing on future SWSs in China. The main research findings are as follows:

1) Both models can effectively reproduce the climatological spatial distribution of observed SWSs, showing high values areas over the Qinghai-Tibet Plateau, the southeast coast, and the northeast and northwest regions. Although CanESM2 simulates slightly stronger SWSs compared to CESM.
2) For CanESM2, external forcing leads to an increase in DJF and JJA SWSs in the eastern China during the historical period, while internal variability plays a notable role in the trend of SWSs changes in the western China. However, for CESM, external forcing only affects the increase in SWSs in the central and eastern regions in JJA.
3) In the future, results from both models indicate that external forcing leads to an increase in winter SWSs in eastern China, while SWSs decreases in the southeastern coastal areas and southwestern Tibet. In summer, SWSs exhibits a pattern of decrease in the north and increase in the south. The magnitude of wind speed changes is greater in winter than in summer. Additionally, as the projected period extends, the magnitude of these changes intensifies.

### 4.2. Discussion

Currently, extensive research has been conducted on future trends in changes to SWSs; however, the underlying causes of these changes remain insufficiently understood [33,34]. Potential contributing factors include modifications in atmospheric circulation and weather system intensity driven by global warming, changes in land cover, and urbanization-induced variations in surface roughness. Nevertheless, the exact extent of their respective impacts remains uncertain [35]. This study offers a preliminary investigation into the future SWSs in China, emphasizing the role of internal variability. Future research should delve deeper into the specific mechanisms and factors driving these changes. Our conclusions have revealed that, based on the results from the two large ensemble simulations, future SWSs changes over China are largely driven by external forcing. Understanding how external forcing leads to regional SWSs variations will be a key focus of our future research efforts.

The projections of regional SWSs changes still carry substantial uncertainty, which primarily stem from three sources: model responses, emission scenarios, and internal variability [36,37]. Statistical or dynamical downscaling methods can effectively enhance the reliability of projections at the regional scale [19,38]. However, differences persist among various downscaling approaches. To reduce inter-model uncertainties, techniques such as multi-model weighting can be employed [39]. Recently, emergent constraint methods based on physical relationships have shown promise in reducing the inter-model uncertainties [40]. Nevertheless, the application of such methods to constrained projections of regional SWSs remains relatively rare. In addition, reducing uncertainties arising from internal variability remains a big challenge for regional projections [41,42].

Assessing wind energy resources is a critical area of research. While previous studies have primarily concentrated on projecting future SWSs in specific regions, variations in wind power are not solely determined by changes in SWSs. Comprehensive wind energy resource assessments must also account for factors such as effective SWSs, the frequency of high wind events, wind energy density, and stability [43]. Future research should prioritize projecting wind energy resources in the context of climate change, emphasizing several key aspects: understanding the impacts of climate change on wind energy potential [44], evaluating the economic feasibility of wind energy development [45], assessing the influence of typhoons and extreme weather events on wind power generation [46], and identifying suitable land areas for wind power installations [47]. These considerations are essential for guiding sustainable wind energy development and optimizing its integration into energy systems.
